# Phonon blockade in a nanomechanical resonator quadratically coupled to a two-level system

**DOI:** 10.1038/s41598-019-45027-z

**Published:** 2019-06-19

**Authors:** Hai-Quan Shi, Xun-Wei Xu, Nian-Hua Liu

**Affiliations:** 1 School of Materials Science and Engineering, Nanchang University, Nanchang, 330031 China; 2 Department of Applied Physics, East China Jiaotong University, Nanchang, 330013 China; 3 Institute for Advanced Study, Nanchang University, Nanchang, 330031 China

**Keywords:** Quantum optics, Microresonators

## Abstract

We investigate phonon statistics in a nanomechanical resonator (NAMR), which is quadratically coupled to a two-level system, by driving the NAMR and two-level system simultaneously. We find that unconventional phonon blockade (UCPNB), i.e., strong phonon antibunching effect based on quantum interference, can be observed when driven fields are weak. By increasing the strengths of the driving fields, we show the crossover from the UCPNB to the conventional phonon blockade (CPNB), which is induced by the strong nonlinear interaction of the system. Moreover, under the strong coupling condition for CPNB, quantum interference effect can also be used to enhanced the phonon blockade by optimizing the phase difference of the two external driving fields.

## Introduction

Phonon blockade^1^ is a quantum effect for preventing the excitation of more than one phonon in a nanomechanical resonator (NAMR), which provides us an effective way to generate single phonons. For the potential application in phononic quantum information processing^2–4^, phonon blockade has draw more and more attentions in recent years^5–18^. The various proposals for realizing phonon blockade so far can be classified into two types namely conventional phonon blockade (CPNB)^1,5–14^ and unconventional phonon blockade (UCPNB)^8,15–18^.

The mechanism for CPNB is attributed to the strong nonlinearity in the system^1^. The strong nonlinearity results in the enharmonic energy level in system, thus the second phonon cannot be excited for the large detuning. Specifically, the strong nonlinearity for mechanical mode can be induced by dispersive (far off-resonant) NAMR-qubit coupling^1,5–7^, a NAMR resonant coupled to a qubit^8^ or a two-Level defect^9^, quadratically optomechanical coupling^10–13^, and the coupling between nitrogen-vacancy (NV) centers and a mechanical mode^14^.

Different from the CPNB, UCPNB is the counter-intuitive phenomenon that strong phonon antibunching can be observed with weak nonlinearity^8,15–18^. Physically, the strong phonon antibunching for UCPNB is based on the destructive interference between different paths for two-phonon excitation^8^, that UCPNB is usually realized by coupling an auxiliary system to the mechanical mode. Recently, UCPNB was predicted in many different systems, e.g., resonant coupled NAMR-qubit system^8^, coupled nonlinear mechanical resonators^15,16^, quadratically optomechanical system^17^, and hybrid optomechanical system^18^.

In this paper, we propose to observe phonon blockade with a quadratically coupling between a NAMR and a two-level system (TLS). The quadratically coupling between a NAMR and a TLS provides us an effective way to generate two phonons at one time^19,20^. We note that the phonon blockade by the quadratically coupling between a NAMR and a TLS has been studied in a recent work^7^. However, different from the previous study^7^, we will focus on the crossover from the UCPNB to CPNB and discuss the phonon blockade induced by the combination of quantum interference effect and strong nonlinearity of the system, which have not been revealed in previous works.

## Results

### Theoretical model

**Figure 1:**
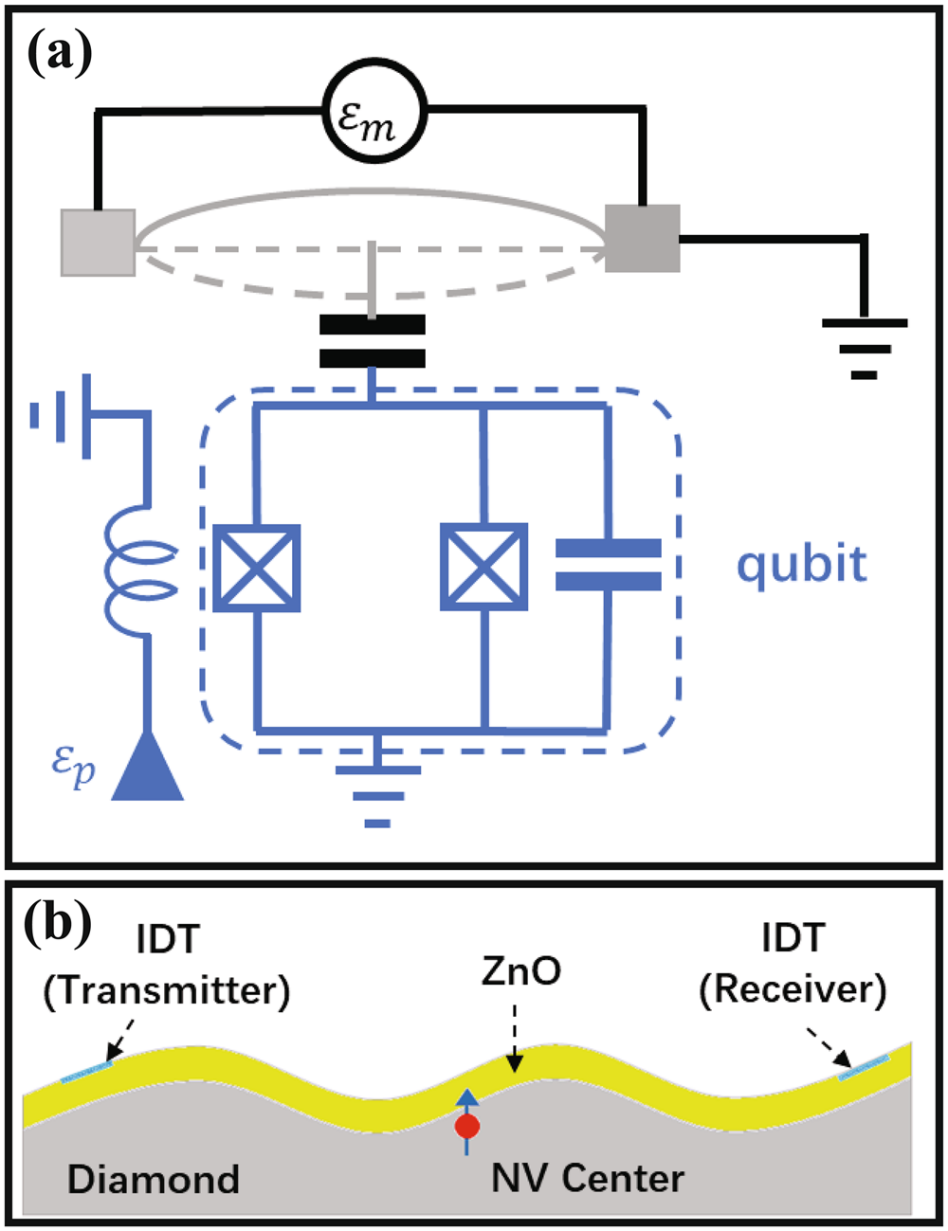
The schematic sketch of (**a**) a nanomechanical resonator coupled to a superconducting qubit^19,25^, (**b**) a phononic crystal with the NV center ensembles located near the surface^14,20,26^.

In this paper, we shall investigate a system in which a nanomechanical resonator is quadratically coupled to a TLS. As shown in Fig. 1, the quadratically coupling between NAMR and TLS can be implemented in a superconducting NAMR-qubit system^19^ [Fig. 1(a)], or in a phononic crystal with NV centers located near the surface^14,20^ [Fig. 1(b)]. We assume that the NAMR is driven by a mechanical pump with amplitude *ε*_*m*_ and frequency *ω*_*b*_ and the TLS is driven by an external field with the strength *ε*_*p*_ and frequency *ω*_*d*_, respectively. The Hamiltonian for the system in the rotating reframe with respect to

$$R(t)=\exp (i{\omega }_{b}{b}^{\dagger }bt+i{\omega }_{d}{\sigma }_{+}{\sigma }_{-}t)\,{\rm{is}}\,{\rm{given}}\,{\rm{by}}\,(\hslash =1)$$

1 $$H=2{\rm{\Delta }}{\sigma }_{+}{\sigma }_{-}+{\rm{\Delta }}{b}^{\dagger }b+J({\sigma }_{-}{b}^{\dagger 2}+{\sigma }_{+}{b}^{2})+({\varepsilon }_{m}{e}^{-i\theta }{b}^{\dagger }+{\varepsilon }_{p}{\sigma }_{+}+{\rm{H}}.\,{\rm{c}}.),$$

where *b* and ${b}^{\dagger }$ denote the annihilation and creation operators of the NAMR with frequency *ω*_*m*_; *σ*_+_ and *σ*_−_ are the raising and lowering operators of TLS with the frequency splitting *ω*_0_; we assume that the frequencies satisfy the conditions, *ω*_0_ = 2*ω*_*m*_ and *ω*_*d*_ = 2*ω*_*b*_, and Δ = *ω*_*m*_ − *ω*_*b*_ is the detuning between NAMR and driving field. *θ* is the phase difference between the two external driving fields. *J* is the quadratically coupling strength between the NAMR and TLS. Without loss of generality, *J* is assumed to be real.

To quantify the statistics of the phonons in the NAMR, we consider the equal-time second-order correlation function in the steady state defined by

2 $${g}_{b}^{(2)}(0)\equiv \frac{\langle {b}^{\dagger }{b}^{\dagger }bb\rangle }{{n}_{b}^{2}},$$

where ${n}_{b}\equiv \langle {b}^{\dagger }b\rangle$ is the mean phonon number. The behavior of the system is described by the master equation^21^ for the density matrix *ρ*

3 $$\begin{array}{rcl}\frac{d\rho }{dt} & = & -i[H,\rho ]+\kappa ({n}_{\sigma ,{\rm{th}}}+1)L[{\sigma }_{-}]\rho +\kappa {n}_{\sigma ,{\rm{th}}}L[{\sigma }_{+}]\rho \\  &  & +\,\gamma ({n}_{m,{\rm{th}}}+1)L[b]\rho +\gamma {n}_{m,{\rm{th}}}L[{b}^{\dagger }]\rho ,\end{array}$$

where $L[o]\rho =o\rho {o}^{\dagger }-({o}^{\dagger }o\rho +\rho {o}^{\dagger }o)/2$ denotes a Lindbland term for an operator *o*, *κ* is damping rate of the TLS and *γ* is damping rate of the NAMR; *n*_*σ*,th_ and *n*_*m*,th_ are the mean numbers of the thermal phonons, given by the Bose-Einstein statistics *n*_*σ*,th_ = [exp(ℏ*ω*/*k*_*B*_*T*) − 1]^−1^ and *n*_*m*,th_ = [exp(ℏ*ω*_*m*_/*k*_*B*_*T*) − 1]^−1^ with the Boltzmann constant *k*_*B*_ and the environmental temperature *T*. The second-order correlation function ${g}_{b}^{(2)}(0)$ can be calculated by solving the master equation (3) numerically within a truncated Fock space.

### Numerical results

Generally, the UCPNB can be discriminated from the CPNB by the optimal conditions for phonon blockade. Based on the Hamiltonian given in equation (1), the optimal conditions for UCPNB can be obtained analytically (the derivation is given in the the section of Methods). When *θ* ≠ *Nπ*/2 (*N* is an integer), the optimal conditions for UCPNB are

4 $${{\rm{\Delta }}}_{{\rm{opt}}}=\frac{1}{4\,\tan \,2\theta }(\gamma -\frac{\kappa }{2}\pm \sqrt{{\rm{\Psi }}}),$$

5 $${J}_{{\rm{opt}}}=-\frac{{\varepsilon }_{m}^{2}\,\cos \,2\theta }{{\varepsilon }_{p}\gamma }(\gamma +\frac{\kappa }{2}\pm \sqrt{{\rm{\Psi }}}),$$

where

6 $${\rm{\Psi }}={(\frac{\kappa }{2}-\gamma )}^{2}-2\kappa \gamma {\tan }^{2}2\theta .$$

When *θ* = *Nπ*/2, the optimal conditions for UCPNB become

7 $${{\rm{\Delta }}}_{{\rm{opt}}}=0,$$

8 $${J}_{{\rm{opt}}}=-\,\frac{{\varepsilon }_{m}^{2}\kappa \,\cos \,2\theta }{{\varepsilon }_{p}\gamma }.$$

The optimal detuning for CPNB is Δ_opt_ = 0 for resonant single-phonon driving, which is the same as the optimal detuning for the UCPNB in the special case for *θ* = *Nπ*/2.

**Figure 2:**
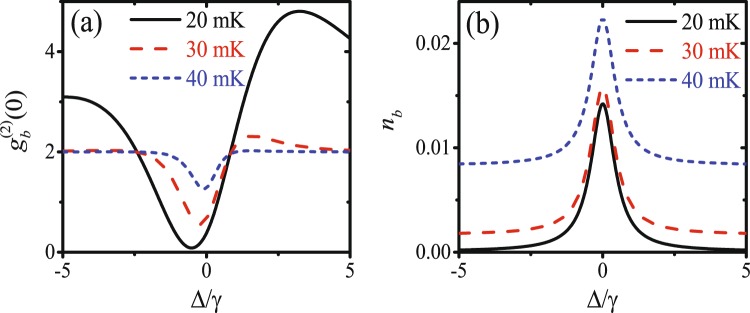
(**a**) The equal-time second-order correlation function ${g}_{b}^{(2)}(0)$ and (**b**) mean phonon number *n*_*b*_ are plotted as functions of the detuning Δ/*γ* for different temperatures: *T* = (20, 30, 40) mK. The other parameters are *ε*_*m*_ = 0.06*γ*, *ε*_*p*_ = 0.06*γ*, *θ* = 0.6*π*, *κ* = 10*γ*, *ω*_0_ = 2*ω*_*m*_ = 2*π* × 8 GHz, and *J* ≈ 0.4*γ* is given by equation (5).

In Fig. 2(a), we show the equal-time second-order correlation function ${g}_{b}^{(2)}(0)$ as a function of the detuning Δ/*γ* with *θ* = 0.6*π* ≠ *Nπ*/2 and *J* ≈ 0.4*γ* given by equation (5). We note that the optimal phonon blockade appears at detuning Δ ≈ −0.52*γ* for *T* = 20 mK, which is in good agreement with the analytical result Δ_o*pt*_ ≈ −0.57*γ* given by equation (4). As equations (4) and (5) are the optimal conditions for UCPNB and the coupling strength is weak (*J* < *γ*), the phonon blockade discovered here should be based on the quantum interference, i.e., the UCPNB. The mean phonon number *n*_*b*_ versus the detuning Δ/*γ* is shown in Fig. 2(b) for different temperatures: *T* = (20, 30, 40) mK. One can also find that the phonon antibunching becomes weaker with the increase of the temperature as well as the thermal phonons.

**Figure 3:**
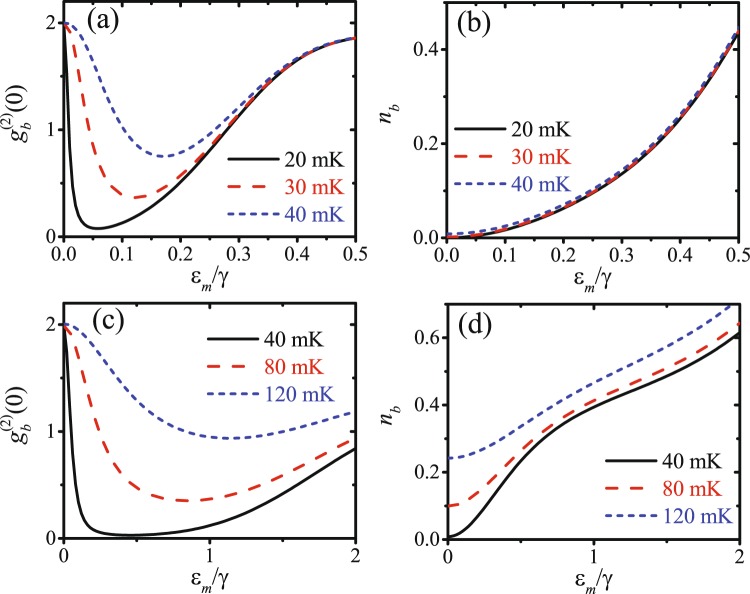
(**a**) ${g}_{b}^{(2)}(0)$ and (**b**) *n*_*b*_ are plotted as functions of the mechanical driving strength *ε*_*m*_/*γ* with optical driving strength ${\varepsilon }_{p}=10{\varepsilon }_{m}^{2}/\gamma$ for different temperatures: *T* = (20, 30, 40) mK. (**c**) ${g}_{b}^{(2)}(0)$ and (**d**) *n*_*b*_ are plotted as functions of the mechanical driving strength *ε*_*m*_/*γ* with optical driving strength ${\varepsilon }_{p}={\varepsilon }_{m}^{2}/\gamma$ for different temperatures: *T* = (40, 80, 120) mK. Δ ≈ −0.574*γ* is obtained from equation (4), and *J* ≈ 0.674*γ* in (**a**) and (**b**), as well as *J* ≈ 6.74*γ* in (**c**) and **(d**) are obtained from equation (5). The other parameters are *θ* = 0.6*π*, *κ* = 10*γ*, and *ω*_0_ = 2*ω*_*m*_ = 2*π* × 8 GHz.

In order to achieve a larger number of mean phonons and improve the robustness against the thermal phonons, we discuss the effect of the driving strengths on the phonon statistics. ${g}_{b}^{(2)}(0)$ and *n*_*b*_ are plotted as functions of the mechanical driving strength *ε*_*m*_/*γ* with optical driving strength ${\varepsilon }_{p}=10{\varepsilon }_{m}^{2}/\gamma$ in Fig. 3(a,b), or ${\varepsilon }_{p}={\varepsilon }_{m}^{2}/\gamma$ in 3(c) and 3(d). At nonzero temperature, the phonon blockade can be enhanced by properly increasing the driving strengths according to the temperature. Moreover, the mean phonon number for phonon blockade in the strong coupling regime with *J* ≈ 6.74*γ* in Fig. 3(d) is much larger than the one in the weak coupling regime with *J* ≈ 0.674*γ* in Fig. 3(b), so that the phonon blockade effect in the strong coupling regime is more robust against than the one in the weak coupling regime.

**Figure 4:**
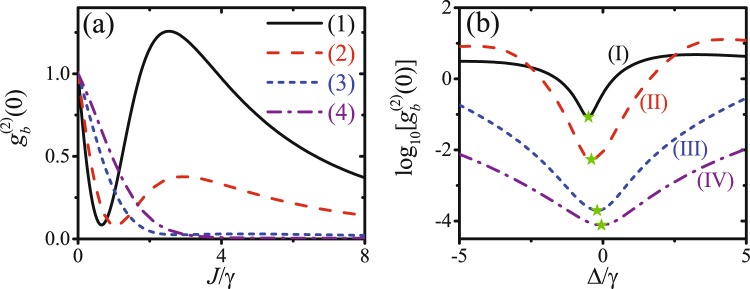
(**a**) ${g}_{b}^{(2)}(0)$ is plotted as a function of the coupling strength *J*/*γ* with detuning Δ given by equation (4) for different mechanical driving strengths: (1) *ε*_*m*_/*γ* = 0.08, (2) *ε*_*m*_/*γ* = 0.1, (3) *ε*_*m*_/*γ* = 0.15, (4) *ε*_*m*_/*γ* = 0.2. (**b**) ${\mathrm{log}}_{10}[{g}_{b}^{(2)}(0)]$ is plotted as a function of the detuning Δ/*γ* with the coupling strength *J* given by equation (5) for different mechanical driving strengths: (I) *ε*_*m*_/*γ* = 0.06, (II) *ε*_*m*_/*γ* = 0.2, (III) *ε*_*m*_/*γ* = 0.4, (IV) *ε*_*m*_/*γ* = 0.6. The other parameters are *ε*_*p*_ = 0.06*γ*, *θ* = 0.6*π*, *κ* = 10*γ*, *ω*_0_ = 2*ω*_*m*_ = 2*π* × 8 GHz, and *T* = 20 mK.

It is worth mentioning that with the enhancing of the mechanical driving strength *ε*_*m*_ as well as the coupling strength *J*, the optimal parameters for UCPNB fail to fit the optimal conditions for phonon blockade. ${g}_{b}^{(2)}(0)$ is plotted as a function of the coupling strength *J*/*γ* with detuning Δ given by equation (4) for different mechanical driving strengths in Fig. 4(a). When the mechanical driving *ε*_*m*_/*γ* is very weak, e.g., *ε*_*m*_/*γ* = 0.08, there is an optimal value of *J* ≈ 0.64*γ*, which is agree with the analytical result *J* ≈ 0.72*γ* given by equation (5) for UCPNB. According to equation (5), the optimal coupling strength *J* for UCPNB increases with the mechanical driving strength *ε*_*m*_. When the mechanical driving *ε*_*m*_/*γ* = 0.2, ${g}_{b}^{(2)}(0)$ decreases monotonically with the coupling strength *J*, which is remarkably different from the optimal coupling *J* ≈ 4.5*γ* given by equation (5) for UCPNB. Moreover, ${\mathrm{log}}_{10}[{g}_{b}^{(2)}(0)]$ is plotted as a function of the detuning Δ/*γ* with the coupling strength *J* given by equation (5) for different mechanical driving strengths in Fig. 4(b). With the increase of the mechanical driving strength, the optimal detuning for phonon blockade is shifted from Δ ≈ −0.52*γ* to Δ ≈ 0, which is also not in accordance with the prediction of UCPNB given by equation (4) for an invariant optimal detuning Δ_o*pt*_ ≈ −0.57*γ*. In fact, when *θ* ≠ *Nπ*/2 (*N* is an integer), the phonon blockade in the strong coupling condition with optimal detuning Δ ≈ 0 is induced by the strong nonlinearity, i.e., CPNB. Figure 4(a,b) show the crossover from the UCPNB to the CPNB by enhancing the mechanical driving strength *ε*_*m*_ as well as the coupling strength *J*.

**Figure 5:**
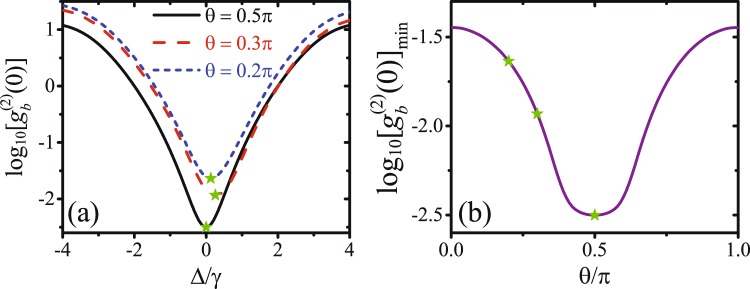
(**a**) ${\mathrm{log}}_{10}[{g}_{b}^{(2)}(0)]$ is plotted as a function of the detuning Δ/*γ* for different phase difference: *θ*/*π* = (0.2, 0.3, 0.5). (**b**) The minimal value of ${\mathrm{log}}_{10}[{g}_{b}^{(2)}(0)]$ by scanning the detuning Δ/*γ* is plotted as a function of the phase difference *θ*/*π*. The other parameters are *J* = 5*γ*, *ε*_*m*_ = 0.2*γ*, *ε*_*p*_ = 0.06*γ*, *θ* = 0.6*π*, *κ* = 10*γ*, *ω*_0_ = 2*ω*_*m*_ = 2*π* × 8 GHz, and *T* = 20 mK.

Lastly, we will shown that, under the strong coupling condition for CPNB, quantum interference effect for UCPNB can also be used to enhanced the CPNB by optimizing the phase difference of the two external driving fields. In Fig. 5(a), ${\mathrm{log}}_{10}[{g}_{b}^{(2)}(0)]$ is plotted as a function of the detuning Δ/*γ* for different phase difference: *θ*/*π* = (0.2, 0.3, 0.5). It is clear that ${g}_{b}^{(2)}(0)$ is dependent on the phase difference *θ*. The minimal value of ${\mathrm{log}}_{10}[{g}_{b}^{(2)}(0)]$ by scanning the detuning Δ/*γ* is plotted as a function of the phase difference *θ*/*π* in Fig. 5(b). Under the strong coupling condition, the minimal value of ${\mathrm{log}}_{10}[{g}_{b}^{(2)}(0)]$ is obtained with *θ* = *π*/2. This can be understated by the fact that, when *θ* = *π*/2, the optimal detunings for UCPNB and CPNB are both Δ = 0, and the CPNB is enhanced by the quantum interference effect for UCPNB.

## Discussion

The direct detection of single phonons is still an outstanding challenge in the present experiments. In some recent experiments^22,23^, the phonon correlation has been measured indirectly by detecting the correlations of the emitted photons from an optical cavity optomechanically coupled to a mechanical mode, which provides us an effective way to investigate phonon statistics experimentally. In addition, indirect phonon detection has also been proposed by the interaction between the mechanical mode and a superconducting microwave resonator^5^ or NV centers^24^.

In summary, we have studied phonon blockade in a NAMR which is quadratically coupled to a TLS. We have shown that UCPNB can be observed in the weak coupling regime based on the destructive interference. In order to increase phonon number and improve the robustness against the thermal noise, we gradually enhanced the driving strengths. We have also shown the crossover from the UCPNB to the CPNB by increasing the mechanical driving strength and the coupling strength. In addition, the CPNB can be enhanced by optimizing the phase difference of the two external driving fields for the combination of quantum interference effect and strong nonlinearity of the system.

## Methods

Assume that the NAMR has been cooled to its ground state, we shall derive the optimal conditions for UCPNB approximately under the weak driving condition {*ε*_*p*_, *ε*_*m*_} < min{*κ*, *γ*}. The wave function can be expanded on a Fock state basis as

9 $$|\psi \rangle ={C}_{g0}|g,0\rangle +{C}_{e0}|e,0\rangle +{C}_{g1}|g,1\rangle +{C}_{g2}|g,2\rangle +\cdots ,$$

where *g* and *e* denote the ground and excited states of the TLS, and *m* represents the Fock state with *m* phonons in the NAMR, and the coefficient |*C*_*gm*_|^2^ (|*C*_*em*_|^2^) is the occupying probability corresponding to the state |*g*, *m*〉 (|*e*, *m*〉). Under the weak driving condition, i.e. {*ε*_*p*_, *ε*_*m*_} < min{*κ*, *γ*}, we will have |*C*_*g*0_| ≫ {|*C*_*e*0_|, |*C*_*g*1_|, |*C*_*g*2_|} ≫ {|*C*_*e*1_|, |*C*_*g*3_|} ≫ …, so the wave function can be truncated to the two-phonon states approximately.

Substituting the wave function in equation (9) and the Hamiltonian in equation (1) into the Schrödinger’s equation *id*|*ψ*〉/*dt* = *H*|*ψ*〉, then the dynamical equations for the coefficients *C*_*gm*_ and *C*_*em*_ are shown as

10 $$\frac{d}{dt}{C}_{e0}=-\,(\frac{\kappa }{2}+i2{\rm{\Delta }}){C}_{e0}-i{\varepsilon }_{p}{C}_{g0}-i\sqrt{2}J{C}_{g2},$$

11 $$\frac{d}{dt}{C}_{g1}=-\,(\frac{\gamma }{2}+i{\rm{\Delta }}){C}_{g1}-i{\varepsilon }_{m}{e}^{-i\theta }{C}_{g0}-i\sqrt{2}{\varepsilon }_{m}{e}^{i\theta }{C}_{g2},$$

12 $$\frac{d}{dt}{C}_{g2}=-\,(\gamma +i2{\rm{\Delta }}){C}_{g2}-i\sqrt{2}{\varepsilon }_{m}{e}^{-i\theta }{C}_{g1}-i\sqrt{2}J{C}_{e0}.$$

In the steady state, i.e. *dC*_*gm*_/*dt* = *dC*_*em*_/*dt* = 0, and under the condition for phonon blockade, i.e. *C*_*g*2_ ≈ 0, we obtain the linear equations for the coefficients *C*_*e*0_, *C*_*g*1_ and *C*_*g*0_ as

13 $$0=-\,(\frac{\kappa }{2}+i2{\rm{\Delta }}){C}_{e0}-i{\varepsilon }_{p}{C}_{g0},$$

14 $$0=-\,(\frac{\gamma }{2}+i{\rm{\Delta }}){C}_{g1}-i{\varepsilon }_{m}{e}^{-i\theta }{C}_{g0},$$

15 $$0=-\,i\sqrt{2}{\varepsilon }_{m}{e}^{-i\theta }{C}_{g1}-i\sqrt{2}J{C}_{e0}.$$

From equations (13 and 14), *C*_*e*0_ and *C*_*g*1_ are given by

16 $${C}_{e0}=\frac{-i2{\varepsilon }_{p}}{\kappa +i4{\rm{\Delta }}}{C}_{g0},$$

17 $${C}_{g1}=\frac{-i2{\varepsilon }_{m}{e}^{-i\theta }}{\gamma +i2{\rm{\Delta }}}{C}_{g0}.$$

Substituting *C*_*e*0_ and *C*_*g*1_ into equation (15), we obtain

18 $$0=(\frac{{\varepsilon }_{m}^{2}{e}^{-i2\theta }}{\gamma +i2{\rm{\Delta }}}+\frac{J{\varepsilon }_{p}}{\kappa +i4{\rm{\Delta }}}){C}_{g0}.$$

As |*C*_*g*0_| ≈ 1 ≠ 0, then we get the conditions for the optimal parameters *J*_opt_ and Δ_opt_ as

19 $${\varepsilon }_{m}^{2}(\kappa \,\cos \,2\theta +4{\rm{\Delta }}\,\sin \,2\theta )+J{\varepsilon }_{p}\gamma =0,$$

20 $${\varepsilon }_{m}^{2}(4{\rm{\Delta }}\,\cos \,2\theta -\kappa \,\sin \,2\theta )+2J{\varepsilon }_{p}{\rm{\Delta }}=0.$$

The optimal parameters for UCPNB given in equations (4–8) are obtained by solving the equations (19 and 20).
